# Using word evolution to predict drug repurposing

**DOI:** 10.1186/s12911-024-02496-1

**Published:** 2024-04-30

**Authors:** Judita Preiss

**Affiliations:** Information School, University of Sheffield, Sheffield, S1 4DP UK

**Keywords:** Drug repurposing, Literature based discovery, Word evolution, Word embeddings, Deep learning

## Abstract

**Background:**

Traditional literature based discovery is based on connecting knowledge pairs extracted from separate publications via a common mid point to derive previously unseen knowledge pairs. To avoid the over generation often associated with this approach, we explore an alternative method based on word evolution. Word evolution examines the changing contexts of a word to identify changes in its meaning or associations. We investigate the possibility of using changing word contexts to detect drugs suitable for repurposing.

**Results:**

Word embeddings, which represent a word’s context, are constructed from chronologically ordered publications in MEDLINE at bi-monthly intervals, yielding a time series of word embeddings for each word. Focusing on clinical drugs only, any drugs repurposed in the final time segment of the time series are annotated as positive examples. The decision regarding the drug’s repurposing is based either on the Unified Medical Language System (UMLS), or semantic triples extracted using SemRep from MEDLINE.

**Conclusions:**

The annotated data allows deep learning classification, with a 5-fold cross validation, to be performed and multiple architectures to be explored. Performance of 65% using UMLS labels, and 81% using SemRep labels is attained, indicating the technique’s suitability for the detection of candidate drugs for repurposing. The investigation also shows that different architectures are linked to the quantities of training data available and therefore that different models should be trained for every annotation approach.

**Supplementary Information:**

The online version contains supplementary material available at 10.1186/s12911-024-02496-1.

## Background

**Fig. 1:**
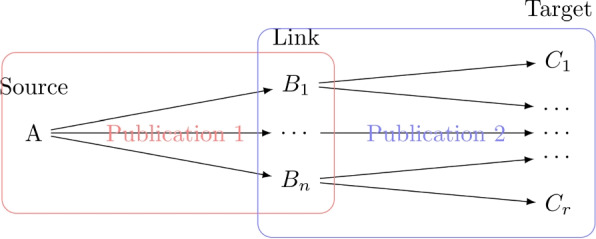
A-B-C literature based discovery: knowledge pairs *A*-$B_1$, ..., *A*-$B_n$, are extracted from publication 1 and $B_1$-$C_1$, ..., $B_n$, ...$C_r$ from publication 2

The development of new drugs is a very long and difficult process [1], often taking up to 10-15 years. The time required for the process can be decreased if an existing, previously tested, drug is being repurposed. Literature based discovery (LBD), which (in its original form) connects knowledge pairs extracted from publications as shown in Fig. 1 [2], has been used previously to suggest possible drug repurposing (e.g. [3]), however, it i) relies on the ability to accurately extract knowledge pairs from publications, ii) frequently generates a large number of candidate knowledge pairs, and iii) omits any knowledge which cannot be extracted as related pairs. Applications of neural networks (NNs) to LBD avoid the first and the last problems, as they can utilise text directly without relying on separate extraction of knowledge pairs. We propose exploring NNs further, specifically i) the use of word embeddings to indicate a drug’s context change prior to it being repurposed, and ii) to evaluate the accuracy of a model based on a time-series of word embeddings used to predict the suitability of a drug for repurposing.

### Literature based discovery approaches

Initial automated approaches to LBD involved inference based on words co-occurring in publication titles (e.g. [4]). However, this approach yielded such high quantities of candidate hidden knowledge pairs that refinements were required to reduce the output. These included more sophisticated relation extraction [5], filtering of the relations used in the inference model and / or the semantic types of the source and target terms (such as only considering relations between treatment and disease) [6]. Distributional semantics based models, which are based on word vectors representing the word’s context, have been shown to outperform traditional approaches when used for e.g. drug/side-effect detection [7].

Other approaches to LBD include knowledge graph based models, which construct a biomedical knowledge graph based on relations extracted from publications and extract new possible paths (e.g. [8]). Recently, machine learning approaches, including deep learning, have been explored further: for example, [9] train a deep learning model on the result of a path ranking algorithm over a knowledge graph built from relations. Most closely related to this work is the use of deep learning to construct vectors representing a word’s context from word definitions and the exploration of the properties of similarity and relation between these word embeddings to yield as yet unobserved hidden knowledge pairs [10].

### Word embeddings

A word embedding, usually learnt from a large quantity of text in a purely unsupervised way, is a vector representing the word’s context within the document collection in a manner which places words with similar meanings close together in the vector space. This feature allows standard operations to be performed on the embedding vectors (denoted below by $\vec {e}$), e.g. [10]:

$$\begin{aligned} \vec {e}(prostate) - \vec {e}(male) + \vec {e}(female) \rightarrow \vec {e}(ovarian) \end{aligned}$$

Word2vec is an efficient technique for learning word embeddings using a shallow neural network [11], with a number of publicly available implementations available. When word embeddings are learnt from data produced across different time periods, changes in word embeddings can be used to detect changes of the word’s use, such as new meanings or semantic shift (e.g. [12]). In this work, we explore whether changes in word embeddings are present prior to a drug being suggested for repurposing.

### Resources

A number of resources, regularly exploited in LBD, are also used in this work:

- *The Unified Medical Language System (UMLS)* [13], a publicly available biomedical methathesaurus, which includes vocabulary standardization for concepts, semantic type information for each concept and a manually created relation file describing relationships between concepts. Mapping words to their UMLS unique identifiers is often the first step of an LBD system.
- *MEDLINE*, the constantly increasing US National Library of Medicine’s collection of biomedical publication abstracts. For a large number of LBD approaches, this forms the basis of their knowledge base.
- *SemRep* [14] and *SemMedDB* [15], a text mining tool which automatically extracts subject-predicate-object triples (such as *Hemofiltration-TREATS-Patients*) from biomedical text. This tool initially maps words to their UMLS unique identifiers and applies manually crafted internal rules to generate the desired triples, which are frequently used as the relations required for LBD. SemMedDB is a SemRep processed version of MEDLINE available for public download (version semmedVER43_2022_R is used in this work).

### Novel contribution

We hypothesise that changes in a drug’s word embeddings may be detectable over time prior to a new potential application of the drug, as (for example) a new body response is observed, and therefore that evolving word embeddings can be used to suggest potential drugs for repurposing investigation. It is important to note that this technique will not necessarily yield the same drugs for repurposing as traditional LBD, as it requires neither the connection between a drug and a body response nor the body response to condition to be overtly present and easy to extract accurately in the document collection.

## Results and discussion

To allow changes in word embeddings to be explored, a large collection of biomedical publication abstracts, MEDLINE, is used to create bi-monthly word embeddings for all drugs appearing in the collection (subject to a minimum frequency requirement). The hypothesis states that a predictable change in word embeddings can be seen before a repurposing. To test this hypothesis, a time series of word embeddings, along with repurposing annotation provided based on UMLS or SemRep, is used to train a deep learning classifier, resulting in a model capable of predicting a drug being suitable for repurposing (see Additional file 1 for a pictorial representation of the pipeline). This novel method of setting up the task allows a large scale 5-fold cross validation evaluation to be performed, avoiding the need to use the small datasets available for LBD evaluation. The steps followed to yield this model follow.

### Word embeddings

**Fig. 2:**
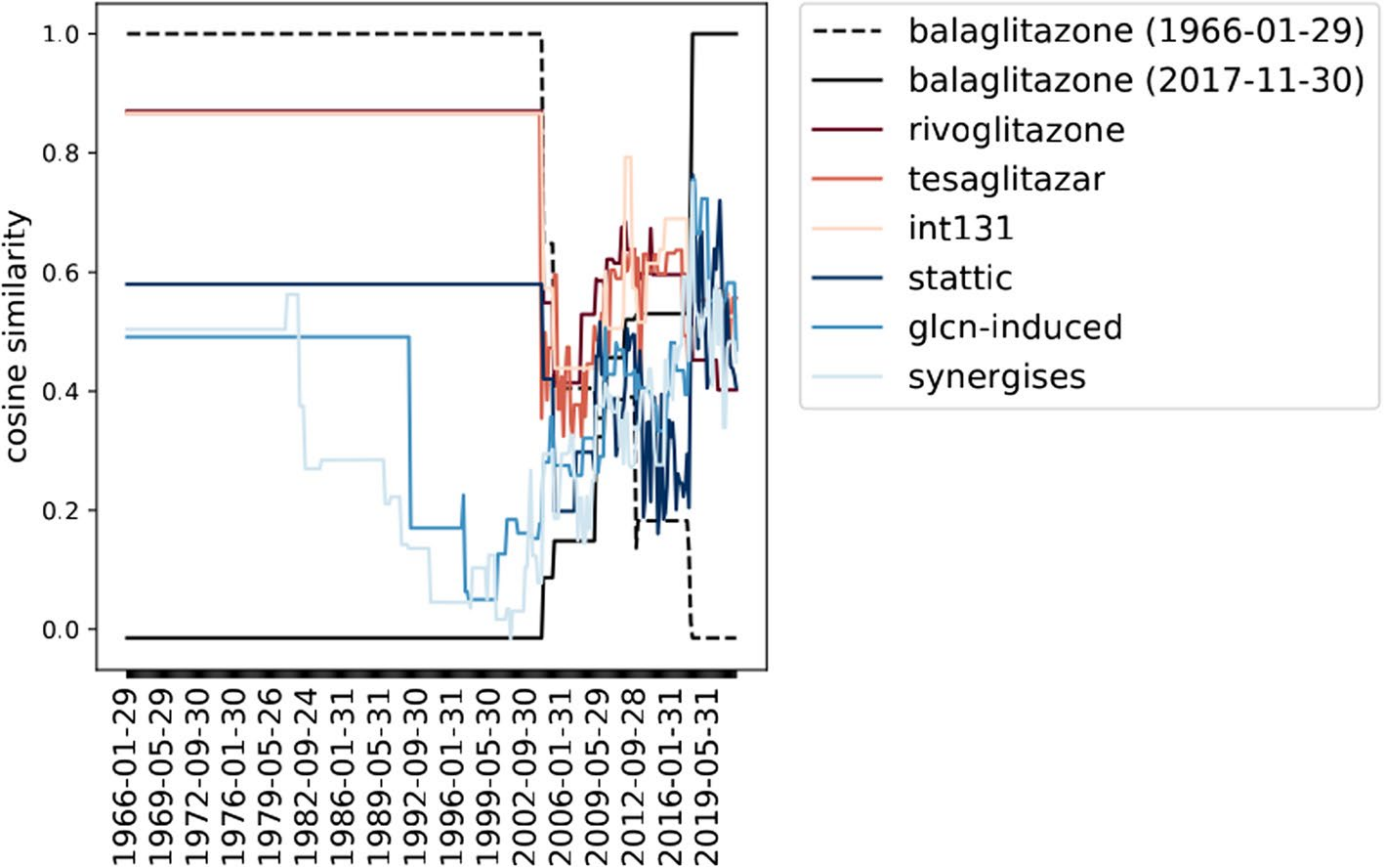
Cosine similarity between the evolving embedding for the drug *balaglitazone* and its nearest neighbours over time; the embeddings were constructed from 1966-2020 abstracts

Abstracts are included for the majority of publications listed in MEDLINE, alongside a date of publication. This allows a chronologically ordered dataset to be created, listing abstracts with their publication dates in increasing order, which can be used to learn word embeddings. In this way, word embedding vectors representing each word’s context at numerous time periods are obtained. Figure 2 shows the cosine similarity between the evolving embedding for the drug *balaglitazone* and its nearest neighbours: the word embedding in 1966 is represented by a dotted black line and the word embedding in 2020 is a solid black line. The change of cosine similarities of these word embedding vectors to the nearest neighbours indicate that a change has taken place during this time period. The change of cosine similarity along the time (x) axis represents the inceptive drift, the change of that time point’s word embedding against the word embedding at the time *t* (for the dotted line, $t=1966$), further demonstrating that the vector representation of the word itself underwent change. (Note that many x-axis labels are not shown in Fig. 2 to preserve readability – the embeddings were computed at bi-monthly intervals.)

An efficient implementation, requiring only a single pass over the entire dataset (rather than separate passes over datasets created for each pre-defined time interval) was used [16]. The associated code^1^ was modified to enable multiprocessing and to avoid reading all data into memory, invoking the word2vec gensim implementation [17] with window size 5, minimum frequency 50 and 5 epochs. The length of embedding vectors was set to 50. Snapshots were taken at bi-monthly intervals starting from 31 November 1965, yielding 336 snapshots in total with 31 November 2021 being the last. By default, embeddings are generated for all words in the document collection (subject to the minimal frequency requirement). However, this work focuses on treatments only, therefore the snapshots were filtered to only include words identified as “clinical drug” (i.e. having the UMLS semantic code T200), resulting in word embeddings for 7,157 distinct clinical drugs.

### Training data

To be able to determine whether a drug is likely to be repurposed, annotated data – i.e. points in time when a novel use for a drug was found – needs to be gathered. Two sources of this information are explored, the first based on UMLS and the second on SemRep extracted triples.

#### UMLS

As mentioned above, the UMLS includes a number of additional files, including a manually created file listing relationships between concepts, such as “*alosetron hydrochloride* may treat *irritable bowel syndrome*”. There has been an average of two releases a year since 2002, with new releases containing revisions and additions over the previous. Assuming the appearance of a new relationship for an existing drug in a new UMLS release signifies its repurposing, extracted relationship pairs can be used to create labels. Specifically, following the extraction of all *treat* and *prevent* relationship pairs from each release of UMLS, the date a new triple appears can be noted. This date can be mapped to one of the embedding snapshot dates, providing a link between the repurposed drug and the corresponding word embedding. Since UMLS versions are produced twice a year, only word embeddings produced at these times are considered with this labelling method.

#### SemRep

As stated, SemMedDB is a publicly available release of SemRep triples extracted from the whole of MEDLINE – i.e. the semantic triples extracted from all abstracts in MEDLINE. Similarly to the UMLS relationships, the relationships between concepts produced by SemRep use a restricted number of predicates including *treats*, *affects* and *prevents*. Each automatically derived relationship is listed alongside the publication identifier of the source abstract, allowing a mapping to the date of publication. It is therefore possible to use a similar approach to above: arranging drug-condition relationships in date order and observing any new drug-condition being added for each drug to detect repurposing instances. In this case, publication dates are continuous so all available embedding vectors can be annotated.

### Repurposing prediction

With a time series of word embeddings for each drug, and an ability to annotate each time instance with respect to the drug’s new usage, prediction of drug repurposing can be set up as a classification problem. Each training instance for a word contains a number of the word’s consecutive word embedding vectors and a binary value based on the label source representing whether the final state (only) is deemed to be an instance of drug repurposing or not (i.e. whether a new relationship was added at the final time). For example the training data for SemRep based annotation, the window, whose size is a hyperparameter (|*w*|), of consecutive bi-monthly word embeddings (*e*) for times *t* to $t+|w|$ for each word ($1 \dots n$) is presented to the algorithm with a label assigned based on the evaluation dataset as follows:

$$\begin{aligned} e1_{t}, e1_{t+1}, e1_{t+2}, ... e1_{t+|w|},{} & {} 1\\ e2_{t}, e2_{t+1}, e2_{t+2}, ... e2_{t+|w|},{} & {} 0\\ \dots{} & {} \\ en_{t}, en_{t+1}, en_{t+2}, ... en_{t+|w|},{} & {} 0\\ \end{aligned}$$

In this example, the word *e*1 shows repurposing in its final state ($t+|w|$), indicated by 1 in the final column. Words *e*2 and *en* do not show repurposing in their final states (shown by a 0). For the example introduced earlier, *balaglitazone*, assuming 2017-11-30 represents its only repurposing, 1 would be present at $t+|w| = 2017\text {-}11\text {-}30$, while the previous states would have the label 0. It is the 0/1 label which will be predicted by the system. Since it is not clear how long prior to repurposing a word’s embedding may show change, the optimum size of the window *w* is explored.

A 5 fold stratified cross validation is performed for each hyperparameter combination to ensure validity and significance of the results. The keras python library [18] is used to explore possible architectures and hyperparameter settings. The explored layers include Long Short Term Memory (LSTM), Bi-directional Long Short Term Memory (BiLSTM), 1D convolution layer (Conv1D), Gated Recurrent Unit (GRU), Simple Recurrent Neural Network (SimpleRNN) and Dropout with varying layer combinations, numbers and sizes. Early stopping was employed with patience 10 to speed up hyperparameter optimization.

### Evaluation

While the UMLS contains preferred versions of concept names, regular expressions were sometimes needed to reduce these to base form (for example, mapping *0.05 ml ranibizumab* to *ranibizumab*) to give access to as many repurposed drugs as possible. However, only 3,840 drugs were found in UMLS with this property and overlapping with the drugs for which word embeddings were acquired (i.e. words with frequency more than 50 in MEDLINE) reduced the dataset further. Using a balanced number of examples gave rise to 1,410 training examples based on the UMLS dataset. The small number of UMLS examples governed the decision behind a 5 fold (rather than 10 fold) cross validation. The test portion therefore corresponded to 20% of the original data with 10% of the training portion dedicated to validation. The training, test and validation sets were stratified, ensuring equal distribution of positive and negative examples across the three subsets. Note that the test portion either represents a publication mentioning that a known drug treats a (previously unconnected) disease (SemRep), or the integration of the drug into UMLS as treating a (previously unlinked) disease, therefore finding the system suggesting a repurposing prior to the publication / UMLS release supports the system being correct in suggesting drugs which should be investigated for repurposing.

A balanced SemRep training corpus gives rise to 20,849 positive and negative instances. While UMLS annotations can only be provided for embeddings at 6-monthly intervals (due to UMLS release frequency), SemRep allows for more frequent annotation of embeddings: bi-monthly intervals were chosen to keep the embedding size within resource processing abilities (bi-monthly embeddings yield a 35GB pickle file). The start year for both evaluations is 2006, dictated by the earliest installable release of UMLS. A stratified 5-fold cross validation, with the number of epochs set to 100, is performed over the hyperparameters which include: batch size, layer types, number and sizes and the maximum history length. Note that the history length allows the optimization step to automatically select a larger embedding interval by ignoring embeddings at specific interval points.

**Fig. 3:**
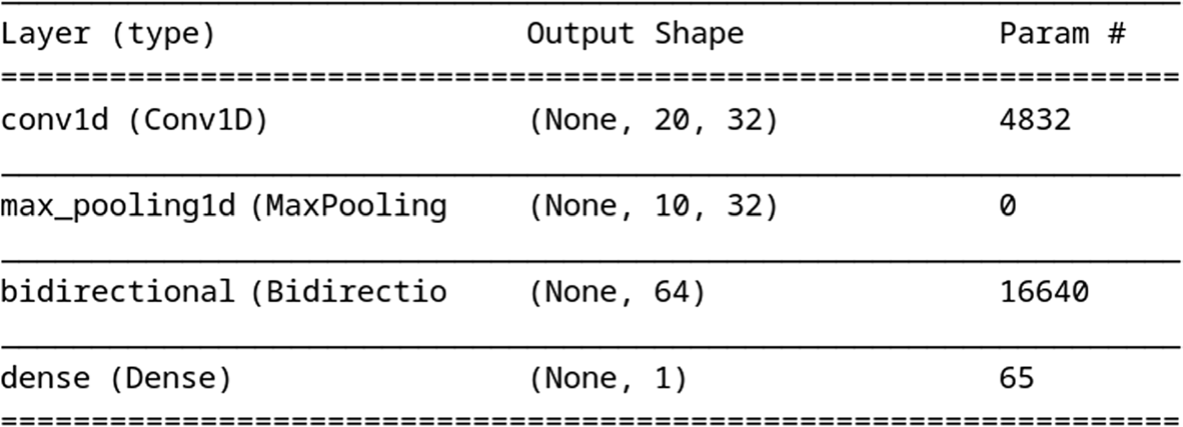
Highest performing SemRep architecture

Combinations of all layer types introduced in the previous sections are explored and the top three results, with their architectures and associated hyperparameter settings, for both label types are presented in Table 1, with the top performing SemRep architecture also shown in a more traditional form in Fig. 3. The results column contains the average accuracy across the 5 folds on the held out test data (whose baseline is 50%). While there is no significant difference between the top three similar architectures for each label type, the architecture for UMLS labels and SemRep labels involve different layers: the relative success of the GRU layer for the UMLS labelled data may be due to the lower number of parameters needing to be trained – while a GRU layer is similar to LSTM, the small quantity of data available for this label type means architectures including this layer type perform significantly worse than the GRU layer based architectures. The use of dropout layers is common across both label types, with suitability supported by their tendency to reduce over fitting. The success of the convolution layer with BiLSTM is supported by their previous success in text classification (e.g. [19]).

**Table 1:** The results for the top three hyperparameter settings: *label* denotes the source of the label of the last column, length is the length of the time sequence employed in training, *L1* and *L2* represent the type of layers 1 and 2 along with their sizes with *D1* and *D2* the size of the intervening dropout layers. If a pooling layer followed L1, its size appears under the column *pool*. The batch size is listed in *batch*

Label	Length	L1	Pool	D1	L2	D2	Batch	Result
UMLS	16	GRU 40	–	0.2	GRU 24	0.2	128	65.04
UMLS	8	GRU 48	–	0.2	GRU 40	0.2	128	64.89
UMLS	12	GRU 32	–	0.2	GRU 32	0.2	64	64.61
SemRep	20	Conv1D 32	2	0.2	BiLSTM 32	0.2	64	81.32
SemRep	20	Conv1D 40	4	0.2	BiLSTM 32	0.2	128	81.31
SemRep	20	Conv1D 62	2	0.2	BiLSTM 40	0.2	64	81.28

Given a sequence of word embedding vectors for a selected drug, the models predict whether the drug should be explored for repurposing. While the performance using either labelling approach exceeds the 50% baseline, the performance of the UMLS labels is significantly lower than that of the SemRep labels. Due to its manual creation, the UMLS labels – while probably more accurate than the automatically derived SemRep labels – are likely to be suffering from errors of omission, resulting in potentially correctly predicted repurposing not being rewarded in the evaluation step. The UMLS performance can therefore be viewed as a type of lower bound.

## Conclusions

We hypothesize that the textual context of a drug changes when a new effect (e.g. body response) is observed, and therefore that word embedding time series can be used to predict drugs worthy of examining for their repurposing potential. Bi-monthly word embeddings are generated from MEDLINE abstracts and a deep learning classifier, determining whether the final embedding in the series has repurposing potential, is built. Two sources of labels indicating repurposing are explored: based on 1) UMLS relations, and 2) SemRep extracted triples. Using a 5-fold cross validation, the UMLS labels yield a 65% accuracy on a balanced test set despite a small quantity of training data. Using the same cross validation, accuracy rises to 81% when SemRep labels are employed. The resulting model can be used on a time series of word embeddings for any drug to predict its suitability for repurposing investigation.

An increase in performance may be possible by mapping drug names to their components: for the purposes of this work, drug concentrations are not considered important and more systematic merging of these may produce a cleaner training set. Similarly, synonyms of diseases may be causing more repurposing observations than is accurate, when for example *fish oil* TREATS *Raynaud phenomenon* appears in known relations and a new relation, *fish oil* TREATS *Raynaud disease* is observed suggesting a repurposing of *fish oil*.

Exploiting full texts of publications for the creation of word embeddings may also yield an improvement: a body response to a drug may be discussed repeatedly in different contexts in the body of a paper, leading to a different embedding.

Using deep learning makes the approach a black box: explainability approaches may reveal importance of a significant embedding position (such as 2 time intervals prior to change) but further insight into a more concrete embedding change leading to repurposing would require significantly more data.

## Methods

The work explores using word evolution to indicate a drug’s suitability for repurposing.

Timeseries of bi-monthly embeddings for each word are built from chronologically ordered publications listed in MEDLINE, and experiments investigate different 1) gold standards indicating repurposed drugs (UMLS or SemRep), 2) window sizes of word embeddings (number of consecutive word embeddings for each word to be used from its acquired word embedding timeseries), 3) architectures (type and quantity of layers) and 4) hyperparameters such as batch size.

A 5-fold cross validation on a balanced dataset is used to obtain an average accuracy of each approach.

### Supplementary Information

**Additional file 1.** Diagram of the classifier pipeline.

## Data Availability

The scripts and a README for the pipeline used in this work are available at https://github.com/juditapreiss/evolution_for_repurposing.

## Abbreviations

BiLSTM: Bi-directional Long Short Term Memory
Conv1D: 1D convolution layer
GRU: Gated Recurrent Unit
LBD: Literature based discovery
LSTM: Long Short Term Memory
NN: Neural network
SimpleRNN: Simple Recurrent Neural Network
UMLS: Unified Medical Language System

## Footnotes

1 Available from https://github.com/cod3licious/evolvemb
