# Supplementary material for: Using word evolution to predict drug repurposing
Source: BMC Med Inform Decis Mak. 2024 Apr 30;24(Suppl 2):114. doi: 10.1186/s12911-024-02496-1 (PMC11061931; doi:10.1186/s12911-024-02496-1)
Supplement: Supplementary file 1 — Additional file 1. Diagram of the classifier pipeline. [file 12911_2024_2496_MOESM1_ESM.pdf]

windowed word embeddings

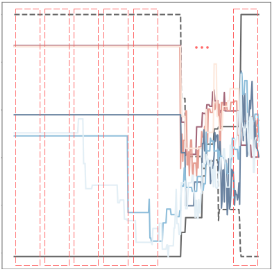

label

amoxicillin,2006-01-01
amoxicillin,2007-01-01
cortisone,2008-06-01
erythromycin,2006-01-01
fish oil,2008-06-01
fish oil,2013-06-01
fish oil,2018-01-01
...

train / eval data: windows of embeddings with labels

deep learning classifier

Layer (type)	Output Shape	Param #
conv1d (Conv1D)	(None, 20, 32)	4832
max_pooling1d (MaxPooling	(None, 10, 32)	0
bidirectional (Bidirectio	(None, 64)	16640
dense (Dense)	(None, 1)	65
